# The impact of school uniforms on primary school student’s physical activity at school: outcomes of a cluster randomized controlled trial

**DOI:** 10.1186/s12966-021-01084-0

**Published:** 2021-01-23

**Authors:** Nicole Nathan, Nicole McCarthy, Kirsty Hope, Rachel Sutherland, Christophe Lecathelinais, Alix Hall, Cassandra Lane, Stewart Trost, Sze Lin Yoong, Luke Wolfenden

**Affiliations:** 1 Hunter New England Population Health, Hunter New England Area Health Service, Newcastle; Locked Bag No. 10, Wallsend, NSW 2287 Australia; 2 School of Medicine and Public Health, The University of Newcastle, University Drive, Callaghan, NSW 2308 Australia; 3 Priority Research Centre for Health Behaviour, The University of Newcastle, University Drive, Callaghan, NSW 2308 Australia; 4 Hunter Medical Research Institute, 1/Kookaburra Circuit, New Lambton Heights, NSW 2305 Australia; 5 Institute of Health and Biomedical Innovation at Queensland Centre for Children’s Health Research, Queensland University of Technology, Brisbane, Australia

**Keywords:** Schools, Uniform, Policies, Girls, Physical activity

## Abstract

**Background:**

Many school-based physical activity (PA) interventions are complex and have modest effects when delivered in real world contexts. A commonly reported barrier to students’ PA, particularly among girls, are uniforms that are impractical (e.g. tunic/dress and black leather shoes). Modifying student uniforms may represent a simple intervention to enhance student PA. The primary aim of this trial was to assess the impact of a PA enabling uniform intervention (shorts, polo shirt and sports shoes) on girls’ moderate-to-vigorous physical activity (MVPA) and total PA i.e. counts per minute (cpm).

**Methods:**

A cluster randomized controlled trial was undertaken in 42 primary schools in New South Wales, Australia. Schools were randomized on one school day to the intervention group, where students wore a PA enabling uniform (their sports uniform) or a control group, where students wore their usual traditional uniform. Student PA was measured using wrist-worn Actigraph GT3X and GT9X accelerometers. Linear mixed models controlling for student characteristics were used to examine the effects of the intervention..

**Results:**

Of the 3351 eligible students, 2315 (69.1%) had parental consent and 2180 of these consenting students participated (94.2%) of which 1847 (84.7%) were included in the analysis. For the primary aim the study found no significant differences between girls at schools allocated to the intervention relative to the control on change in MVPA (0.76 min, 95% CI − 0.47 to 1.99, *p* = 0.22) or cpm (36.99, 95% CI − 13.88 to 87.86, *p* = 0.15). Exploratory analysis revealed small effects for a number of findings, including significant reduction in sedentary activity (− 1.77, 95% CI − 3.40 to − 0.14, *p* = 0.035) among all students at schools allocated to the intervention, and non-significant improvements in girls’ light intensity PA (1.47 min, 95% CI − 0.06 to 3.00, *p* = 0.059) and sedentary activity (− 2.23 min; 95% CI − 4.49 to 0.02, *p* = 0.052).

**Conclusion:**

The findings suggests that the intervention may yield small improvements in some measure of PA and require substantiation in a larger RCT with longer-term follow-up. The inclusion of additional intervention components may be required to achieve more meaningful effects.

**Trial registration:**

The trial was prospectively registered with Australian New Zealand Clinical Trials Register ACTRN12617001266358 1st September 2017.

**Supplementary Information:**

The online version contains supplementary material available at 10.1186/s12966-021-01084-0.

## Background

Children’s participation in at least 60 min of moderate-to-vigorous physical activity (MVPA) per day is essential for their healthy growth and development [1] and the prevention of future chronic disease [2]. Despite this, international research indicates that many school-aged children, girls in particular, are not sufficiently active [3]. Girls are between 17 and 19% less active than boys [4, 5], with differences beginning from as young as 8 years [5, 6]. As such, improving physical activity during childhood, particularly among girls, has been identified as a public health priority [7].

Schools are a key setting for the promotion of physical activity as they reach almost all students on an on-going basis [8]. Despite this, school-based interventions to improve girls’ physical activity have reported mixed effects [9, 10]. While efficacious interventions exist, they are often multi-component, comprehensive programs that target physical activity opportunities before, during and after school [11, 12]. The complex nature of multi-component school-based interventions make them susceptible to poor implementation fidelity, particularly when their delivery is attempted in more real world contexts [12]. Further, such interventions demand significant ongoing investment by governments in staff training, infrastructure and resources to deliver and be maintained. Consequently, such interventions are often overlooked by policy makers as credible options for large scale dissemination [13, 14]. Simple interventions that are effective, scalable and low cost are urgently needed if population rates of physical activity among girls are to be improved [15–17].

Many jurisdictions internationally including Australia, United Kingdom, Japan and parts of south-east Asia, Canada, Africa and South America require students in some or all schools to wear a uniform to school [18–20]. Typically, such uniforms are formal, and girls are required to wear a uniform that may comprise of a dress or tunic with socks or stockings and black leather shoes. A reported impediment to girl’s physical activity at school is the impracticality of their uniforms. For example, a 2006 review by Allender et al. [21] which included 24 qualitative research studies of UK children’s and adults’ reasons for participation and non-participation in sport and physical activity, identified that inappropriate uniforms were major barriers to girls participating in school sport. In Australia a qualitative study of 54 primary school children from six schools found that girls reported that their uniform significantly limited their ability to be active at break time, stating uniforms “held them back from running” and restricted them from playing sports [16]. Furthermore there is some evidence to suggest an association with uniforms and girls school activity. For example, a 2012 repeat cross sectional study of 64 Grade 6 students from one Western Australian school, found that girls took significantly more steps during break times when wearing sports uniform (1134.1 steps) compared to their traditional uniform (933.3 steps) (*p* = 0.006) [15].

These findings suggest that girls’ natural tendency for physical activity may be facilitated through a simple change to a physical activity enabling uniform. If found to be effective, such an intervention may have considerable public health appeal particularly as it may be a more sustainable strategy in the long term. As such, it may be more amenable for large scale implementation than many of the complex school-based physical activity interventions that have dominated the literature to date. Previous comprehensive systematic reviews of the literature have failed to identify experimental research examining the effectiveness of interventions to increase girls’ physical activity through uniform modifications. To address this important evidence gap, a study was conducted to assess the impact of a uniform intervention on girl’s aged 8–10 years physical activity levels across the whole school day, as well as during school break time. The secondary aim was to assess the impact of the uniform intervention on students (both girls and boys) aged 8–10 years physical activity levels across the school day as well as school breaks.

## Methods

Approval to conduct this study was obtained from Hunter New England Human Research Ethics Committee (06/07/26/4.04), University of Newcastle (H-2008-0343), and the NSW Department of Education and Maitland-Newcastle Catholic Schools Office.

### Design and setting

The study employed a cluster randomized controlled trial (cRCT) in primary schools (which cater for children aged 5–12 years), in the Hunter New England region of NSW. Schools were the unit of allocation and randomly allocated to receive the intervention or to a no intervention control. Physical activity outcome data were collected at the student level via accelerometer. The Hunter New England region covers a large geographical region (more than 130,000km^2^) and consists of a socioeconomically and demographically diverse population of approximately 117,000 children aged 5–14 years [22] and over 400 primary schools. The study follows the CONSORT guidelines and TIDIER guideline for intervention studies (see Appendix for checklist).

### Participants, recruitment, randomization and blinding

#### Schools

Government and Catholic schools from the study region were placed in a random order and invited to participate. Schools were excluded in they were participating in another physical activity intervention; or catered exclusively for children requiring specialist care; or have a sports uniform that children wear each day. School principals were provided with a study information package and asked to provide written informed consent. Consenting eligible schools were randomly allocated in a 1:1 ratio to the uniform intervention or control by an independent investigator using a computerized random number generator.

#### Students

Following principal consent all children in Grades 2 and 3 (aged 8–10 years) were provided, by a member of the research team, an information package that they were asked to give to their parent or guardian. The information package included an information statement overviewing the purpose of the study and a consent form which sought active consent for their child to participate in the study. Two weeks following distribution of the information packages, parents who had not returned a consent form were telephoned by staff employed through the school and asked if they would like to consent to child participation. As the intervention was initiated school wide, consent from parents and children was sought for participation in the data collection component only.

### Intervention

Both intervention and control schools had a “traditional school uniform”, which for girls consists of a skirt or dress and for boys, shorts and a button up shirt with black leather shoes for both, and a uni-sex sports uniform which consists of the same shorts and polo-shirt for girls and boys which is worn with sports shoes. The usual school uniform is typically worn 4 days per week and the sports uniform once per week on their designated sports day. School staff, supported by the research team, asked all students in Grades 2 and 3 in the intervention arm to wear their school sports uniform on a day when they would normally wear a traditional uniform (i.e. not their scheduled sports day). Sports day, a single day in the week where all children engaged in organised sport and are required to wear a sports (physical activity enabling) uniform were excluded. This enabled a contrast of the effects of wearing versus not wearing an activity enabling uniform on measures of physical activity on usual (non-sport day) days of the week. Thus the activity enabling uniform intervention day was randomly generated by an independent statistician using a computerized random number function in Microsoft Excel (excluding their sports day). This day is referred to as “intervention day” while the remaining days are referred to as “usual days”. There were no other changes to school or classroom timetables or curricula.

### Control schools

Students in control schools continued with their schools’ normal uniform practices during the data collection period. A random non-sports day where students wore their traditional school uniform was picked to serve as the ‘comparison day’.

### Data collection and measures

#### School characteristics

Data regarding school type (Government, non-Government Catholic), number of students and the postcode of the locality of the school were obtained from participating schools.

#### Student characteristics

Students’ sex, age and residential post-code were collected from student consent forms.

#### Primary aim: girl’s mean MVPA and mean physical activity counts per school day

Girls’ physical activity during school hours were measured objectively using wrist-worn ActiGraph GT3X+ and GT9X accelerometers (ActiGraph Corporation, Pensacola, FL). Two research assistants visited each class and demonstrated to students and teachers how to fit the accelerometer to the wrist of their non-dominant hand and answered any questions they may have had. Teachers assisted those students who could not fit the accelerometer themselves. Students wore accelerometers for one school week (Monday through Friday) for the whole school day (9 a.m to 3 p.m), except during water-based activities. To be included in the analysis, students needed to have worn the accelerometer for at least 80% of the intervention day (i.e. sports uniform day) and usual day. Accelerometer non-wear time was calculated by summing the number of consecutive zero counts accumulated in strings ≥20 min. Wear time was estimated by subtracting non-wear time from the total monitoring time for the school day. Accelerometer counts were classified as sedentary, light-intensity physical activity, and MVPA using the vertical axis wrist cut-points developed by Chandler et al. [23] Counts per minute (cpm) was calculated by dividing the total accelerometer counts by the minutes of wear time. Activity across the whole school day as well as segmented for break time (i.e. recess and lunch time) was assessed. Both intervention and control schools were randomly allocated to have data collected in 1 week during a 6 week schedule i.e. from 30th October to 11th December 2017.

#### Secondary aim: all student’s mean MVPA and counts per minute

As per the primary aim the mean MVPA and counts per minute for all students (boys and girls) were assessed across the whole school day as well as segmented for break times.

### Sample size and power

Based on 44 schools (22 per group) with an average of 40 Grade 2–3 female students, assuming a participation rate of 50%, an ICC of 0.05, and an MVPA standard deviation of 12 [11], this would be sufficient to detect an absolute difference in girls’ minutes of MVPA of approximately 3.2 min with 80% power, and an alpha of 0.05.

### Analyses

All analyses were performed in SAS 9.3 (SAS Institute Inc., Cary, NC). Descriptive statistics were used to describe the school (school size, type, geographic location) and student sample (sex, age, grade, geographic location and socio-economic status). School and student postcodes were used to categorize their locality as either ‘rural’ (outer regional, remote and very remote areas) or ‘urban’ (regional cities and inner regional areas) based upon the Australian Bureau of Statistics (ABS) Australian Standard Geographical Classification (ASGC) system. Schools and students whose postcodes were ranked in the top 50% of NSW postcodes, based on the ABS Socio-Economic Indexes For Australia (SEIFA), were categorized as ‘higher socio-economic areas’, while those in the lower 50% were categorized as ‘lower socio-economic areas’. Linear mixed models controlling for students socio-economic area, school type, geographic location, wear time and sex were used to assess the mean change in outcomes, from ‘usual days’ to ‘intervention day’, between the two groups. All models included fixed effects for group, intervention day and a group by intervention day interaction term, as well as a random intercept for school to account for the clustered design of the trial, and a random intercept for child nested within school, to account for the repeated measurements taken on children. For each outcome, sub-group analyses were undertaken to determine the intervention effect within sex, by adding a gender-by-group-by-intervention day interaction term as a fixed effect in the original model. For completeness and for descriptive purposes only, the effect of the intervention on boys was analyzed and presented with that of girls and with the whole sample combined, as well as the differential effect between boys and girls. Additionally, given evidence of the benefits of reduced sedentary activity, and increase of light activity, post-hoc exploratory analyses were undertaken to assess any potential effect of the intervention for these outcomes. Such analyses were not registered a-priori and were intended to be hypothesis generating. Finally, student physical activity data were analysed across the whole school day and segmented for break times. All statistical tests were two tailed and alpha level was set at 0.05.

## Results

**Fig. 1:**
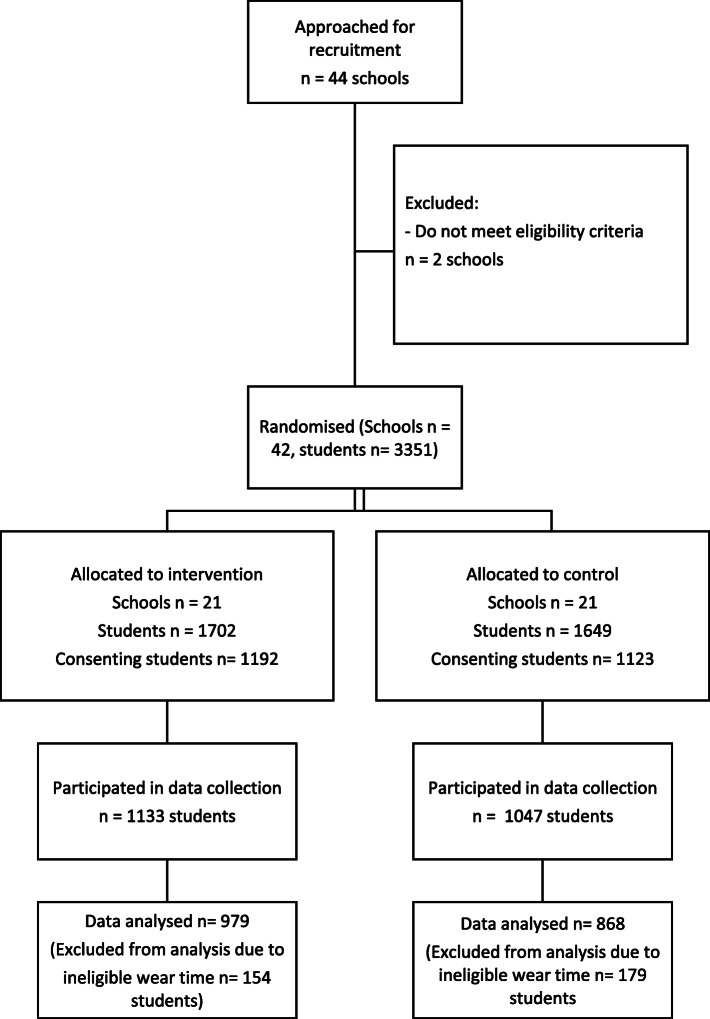
CONSORT diagram

Of the 44 schools that agreed to participate, two schools were deemed ineligible and were excluded (prior to randomization) as they allowed students to wear their sports uniform each school day. This left 42 schools in the final sample. From these schools, overall consent was obtained for 2315 Grade 2–3 students (69.1%) including 1195 girls (48.4% of consenters). Student sex was significantly associated with consent, with slightly more girls consenting than boys (69.3% vs 64.5%). Of these students, 2180 wore an accelerometer (94.2% of consenting students), including 1146 girls (95.9% of consenting girls). Overall, 1847 students and 961 girls that wore an accelerometer provided valid outcome data. (see Fig. 1 CONSORT).

Characteristics of the participating schools and students are outlined in Table 1. A greater proportion of schools allocated to the intervention were from more disadvantaged areas (71% vs 57%) and a higher proportion of students attending intervention schools were from major cities (67% vs 52%). Otherwise the characteristics of schools and students allocated to intervention and control groups were similar.

**Table 1:** Characteristics of schools and participants

Characteristics	Intervention	Control
**School**	*N* = 21	*N* = 21
Mean school size	290.24 (155.18)	294.05 (168.52)
School Type		
Catholic	8	9
Government	13	12
Urban/ Rural Index		
Major cities	10 (48%)	11 (52%)
Inner and outer regional and remote	11 (52%)	10 (48%)
Socio-economic index		
Most disadvantaged	15 (71%)	12 (57%)
Least disadvantaged	6 (29%)	9 (43%)
**Student**	*N* = 979	*N* = 868
Female	501 (51%)	460 (53%)
Mean age (SD)	8.22 (0.70)	8.15 (0.67)
Class		
Grade 2	478 (49%)	475 (55%)
Grade 3	501 (51%)	393 (45%)
Urban/ Rural Index		
Major cities	655 (67%)	448 (52%)
Inner/outer/remote	324 (33%)	420 (48%)
Socio-economic index		
Most disadvantaged	499 (51%)	435 (50%)
Least disadvantaged	480 (49%)	433 (50%)

### Primary aim: girls’ MVPA and counts per minutes during the school day

There was no significant difference between groups for change in girls’ MVPA (0.76 min, 95% CI − 0.47 to 1.99, *p* = 0.22) or cpm (36.99, 95% CI − 13.88 to 87.86, *p* = 0.15) (Table 2). Similarly, when segmented for break time there was no significant impact of the intervention on girls’ MVPA (0.13 min, 95% CI − 0.61 to 0.87, *p* = 0.73) or cpm (35.07, CI − 111.14 to 181.28, *p* = 0.63) (Table 3).

### Secondary aim: all student’s MVPA and counts per minute during the school day

There was no significant difference in students’ (combined boy and girl) change in school day MVPA between groups (0.71 min, 95% CI − 0.18 to 1.60, *p* = 0.11) or cpm (36.41, 95% CI − 0.42 to 73.23, *p* = 0.053) (Table 2). There was no differential effect between females and males for MVPA (0.12 min, 95% CI − 1.65 to 1.90, *p* = 0.89) or cpm (1.96, 95% CI − 71.76 to 75.67, *p* = 0.96) (data not presented in tables). When segmented for break time there remained no significant between group differences on change in MVPA (0.30 min, 95% CI − 0.23 to 0.84, *p* = 0.26) or cpm (94.16, 95% CI − 11.86 to 200.18, *p* = 0.08) (Table 3).

**Table 2:** Between group mean differences in students’ change in physical activity levels during school hours

	1. Intervention		2. Control		Group x Intervention Day Differential effect	
Outcome	Intervention day mean (SD)	Usual day mean (SD)	Comparison day mean (SD)	Usual day mean (SD)	Estimate^**a**^	***p***-value
**Moderate-vigorous physical activity (MVPA)**						
***Girls***	36.87 (SD = 11.49)	36.24 (SD = 12.19)	35.56 (SD = 11.81)	35.57 (SD = 12.00)	0.76 [−0.47; 1.99]	0.22
***Boys***	40.99 (SD = 12.55)	39.76 (SD = 12.11)	40.52 (SD = 12.65)	40.38 (SD = 13.13)	0.64 [−0.65; 1.92]	0.32
***All students***	38.88 (SD = 12.19)	37.94 (SD = 12.27)	37.94 (SD = 12.53)	37.83 (SD = 12.77)	0.71 [−0.18; 1.60]	0.11
**Counts per minute (cpm)**						
***Girls***	2572.00 (SD = 475.56)	2530.32 (SD = 516.98)	2500.05 (SD = 477.19)	2495.40 (SD = 491.03)	36.99 [−13.88; 87.86]	0.15
***Boys***	2820.61 (SD = 522.13)	2764.54 (SD = 516.01)	2783.29 (SD = 524.40)	2759.39 (SD = 537.64)	35.04 [−18.34; 88.41]	0.19
***All students***	2693.38 (SD = 513.86)	2643.55 (SD = 529.51)	2634.83 (SD = 521.72)	2619.25 (SD = 530.37)	36.41 [−0.42; 73.23]	0.053
**Light physical activity**						
***Girls***	115.00 (SD = 17.92)	111.82 (SD = 19.73)	113.18 (SD = 20.65)	111.77 (SD = 18.91)	1.47 [−0.06; 3.00]	0.059
***Boys***	118.69 (SD = 18.81)	115.06 (SD = 19.26)	116.47 (SD = 20.66)	114.81 (SD = 19.11)	0.58 [−1.02; 2.19]	0.47
***All students***	116.80 (SD = 18.45)	113.39 (SD = 19.57)	114.72 (SD = 20.70)	113.18 (SD = 19.05)	1.06 [−0.05; 2.17]	0.06
**Sedentary activity**						
***Girls***	201.31 (SD = 25.62)	204.18 (SD = 28.33)	203.87 (SD = 26.80)	204.01 (SD = 27.08)	−2.23 [−4.49; 0.02]	0.052
***Boys***	193.16 (SD = 25.60)	195.39 (SD = 27.21)	195.02 (SD = 26.65)	198.35 (SD = 26.15)	−1.22 [−3.58; 1.15]	0.31
***All students***	197.33 (SD = 25.92)	199.93 (SD = 28.13)	199.68 (SD = 27.08)	201.34 (SD = 26.81)	−1.77 [−3.40; −0.14]	0.035^*^

^**a**^**controlling for students socio-economic, school type, geographic location, wear time and sex**

### Exploratory analyses: light and sedentary activity

Among girls, exploratory analyses releveled non-significant improvements between groups on measures of change in both light (1.47 min, 95% CI − 0.06 to 3.00, *p* = 0.059) and sedentary activity (− 2.23 min; 95% CI − 4.49 to 0.02, *p* = 0.052) (Table 2). Change in light activity during break times, was significantly higher among girls attending intervention schools (0.62 min, 95% CI 0.15 to 1.10, *p* = 0.012) relative to control schools (Table 3).

**Table 3:** Differential effect in students’ physical activity levels during break time

	Moderate-vigorous physical activity (MVPA)	Counts per minute (cpm)	Light physical activity	Sedentary activity
**Estimate**^**a**^ **(95% CI)** (***p*-value)**				
***Girls***	0.13 (− 0.61, 0.87) (*p* = 0.73)	35.07 [− 111.14; 181.28] (*p* = 0.63)	0.62 [0.15; 1.10] **(*p*** = 0.012)*	−0.75 [− 1.63; 0.13] (*p* = 0.09)
***Boys***	0.50 [− 0.27; 1.28] (*p* = 0.20)	160.56 [6.99; 314.14] **(*p*** = 0.041)	0.38 [− 0.12; 0.88] (*p* = 0.13)	−0.88 [− 1.80; 0.04] (*p* = 0.06)
***All students***	0.30 [− 0.23; 0.84] (*p* = 0.26)	94.16 [−11.86; 200.18] *p* = 0.08	0.51 [0.16; 0.85] **(*p*** < 0.01)*	−0.81 [− 1.45; − 0.17] **(*p*** = 0.014)*

^a^controlling for students socio-economic, school type, geographic location and wear time

For all students combined, exploratory analysis revealed non-significant differences between groups on measures of change in light activity (1.06, 95% CI − 0.05 to 2.17, p = 0.06) and significant differences on measures of sedentary activity (− 1.77, 95% CI − 3.40 to − 0.14, *p* = 0.035) (Table 2). When segmented for break time, however, there were significant differences between groups for both change in light (0.51, 95% CI 0.16 to 0.85, *p* < 0.01) and sedentary activity (− 0.81, 95% CI − 1.45 to − 0.17, *p* = 0.014) (Table 3).

## Discussion

To our knowledge, this is the first randomized controlled trial to assess, via accelerometry, the impact of students’, in particular girls’, school uniforms on their school-day physical activity. The study found no significant differences between groups on the primary aim of change in girls MVPA and total physical activity (cpm) from the usual uniform day to intervention day. However, exploratory analysis found small improvements in light intensity physical activity (1.47, 95% CI -0.06, 3.00, *p* = 0.059) and reductions in sedentary activity (− 2.23, 95% CI -4.49, 0.02, *p* = 0.052) favoring girls in the intervention group, which approached significance. For all students combined, this study found significant reductions, relative to controls among intervention students, in sedentary activity (− 1.77, 95% CI -3.40, − 0.14, *p* = 0.035). Such improvements in light intensity physical activity and sedentary behavior however, are very small in absolute terms and were uncertain.

On measures of MVPA, the effect sizes reported in this trial, while non-significant, appeared higher than previous trials. The most recent meta-analysis of cRCTs with accelerometer-measured outcomes found a standardised mean difference (SMD) in girls MVPA of 0.07 (95% CI: − 0.07,  0.21) across 14 school-based interventions [12]. The effect reported in this trial, were equivalent to a SMD in MVPA of 0.46 (95% CI: − 0.46, 1.39), and represents the second highest point estimate (effect) of all physical activity interventions identified in this recent systematic review. Importantly, the effect size was achieved by an intervention that is likely far less expensive to implement than the complex multicomponent interventions previously trialled. These characteristics make the intervention a potentially attractive candidate for large scale implementation if the effects can be confirmed longer-term in a larger randomized controlled trial.

Surprisingly, there was no significant differential effect of the intervention by gender on MVPA or counts per minute. However, for all students, measures of total physical activity (cpm) approached statistical significance (0.053). The findings suggest that traditional school uniforms impede physical activity among boys and girls and that changes to school uniforms may be beneficial for all students, but that it may not reduce the considerable gap in physical activity between the sexes. Additional intervention strategies that target activity in girls more broadly in the school environment to ensure they have equitable access to space, equipment and that activities focus on enjoyment may be required to achieve this [24].

While undertaken as exploratory analyses the intervention significantly reduced sedentary activity of all students. There did, however, appear to be differential effects of the intervention, on measures of light and sedentary activity. For example subgroup analyses on measures of sedentary activity found the effect size approached significance for girls (− 2.23, 95% CI − 4.49 to 0.02, *p* = 0.052) but was almost twice that reported among boys (− 1.22, 95% CI − 3.58 to 1.15, *p* = 0.31). Given the increase in girls light physical activity the intervention may be successful in facilitating less rigorous play-based activities for girls. Further research, including observational methods that can capture physical activity characteristics and context is required to confirm the effects of the intervention on these outcomes and test this hypothesis. However, the effect sizes reported for both measures of light and sedentary behaviour were very small in absolute terms, and may confer little benefit to individual health status. The inclusion of other intervention components may be required to further enhance the impact of the intervention on these measures.

### Limitations

The findings of the study should be considered in the context of a number of limitations. First, the trial outcomes were assessed based on a one-day intervention. A larger RCT assessing the effects over a longer exposure period, is required to verify the findings of this trial, to allow for any initial reactivity to the uniform changes and to assess sustained effects. Second, the trial only assessed physical activity across the school day. Potentially, increases in physical activity occurring at school may have been offset by some reductions occurring outside of school hours. Conversely the opportunity to wear sports uniform and sports shoes to school may have encouraged some children to utilise active transport to and from school thus having a positive impact on physical activity levels out of school hours. As 24-h physical activity data were not assessed, the extent to which this occurred is unknown and thus should be addressed in a future trial. Third, due to timing and resource constraints we fell short of recruiting our intended sample of 60 schools which would have provided more precision around the effect size of our primary aim. Given the potential for this intervention to be delivered to a large number of schools adequately powered future trials to detect clinically meaningful changes is needed. Fourth, in order for any policy change to be implemented within schools it must have the support of the school community, which was not measured as part of this trial. However studies conducted by the research team suggests that there is overwhelming support from students, in particular girls [25], their parents and teachers [26] for the implementation of sports uniforms only, with many reporting that it would make them more active at school. Finally, the trial was conducted in one region of NSW Australia. The generalisability of the findings to schools in other jurisdictions is limited.

## Conclusion

Girls’ tendency to engage in less physical activity, and higher levels of sedentary activity, may significantly impact on their immediate and long-term physical, social, cognitive and psychological health. Thus, there has been considerable investment in school-based interventions that aim to engage girls and facilitate their physical activity opportunities. The findings of this trial suggest that simple uniform changes, a low cost, easily disseminated intervention, may be capable of achieving small improvements in measures of student physical activity and may contribute to improvements in population level physical activity if implemented as part of more comprehensive public health initiatives.

## Supplementary Information

**Additional file 1.** CONSORT 2010 checklist of information to include when reporting a randomised trial*.

## Data Availability

The datasets used and analyzed during the current study are available from the corresponding author on reasonable request.
